# Mogroside V Improves Follicular Development and Ovulation in Young-Adult PCOS Rats Induced by Letrozole and High-Fat Diet Through Promoting Glycolysis

**DOI:** 10.3389/fendo.2022.838204

**Published:** 2022-03-28

**Authors:** Lan’e Huang, Aihong Liang, Tianlong Li, Xiaocan Lei, Xi Chen, Biyun Liao, Jinru Tang, Xiting Cao, Gang Chen, Fengyu Chen, Yiyao Wang, Linlin Hu, Weiguo He, Meixiang Li

**Affiliations:** ^1^ Department of Histoembryology, Clinical Anatomy and Reproductive Medicine Application Institute, Hengyang Medical School, University of South China, Hengyang, China; ^2^ Reproductive Medicine Center, The Affiliated Hospital of Youjiang Medical University for Nationalities, Baise, China

**Keywords:** mogroside V (MV), polycystic ovary syndrome (PCOS), lactate, glycolysis, follicular development, ovulation

## Abstract

Polycystic ovary syndrome (PCOS) is a heterogeneous endocrine disorder characterized by hyperandrogenism, ovulatory dysfunction, and polycystic ovaries. In this study, we induced a young-adult PCOS rat model by oral administration of letrozole combined with a high-fat diet and then treated with mogroside V (MV) to evaluate the protective effects of MV on endocrine and follicle development in young-adult PCOS rats. MV (600 mg/kg/day) administration not only significantly reduced the body weight and ovary weight, but also attenuated the disrupted estrous cycle and decreased the level of testosterone. MV restored the follicular development, especially by increasing the number of corpus luteum and the thickness of the granular layer in young-adult POCS rats. Moreover, metabolomics showed that MV markedly increased the levels of D-Glucose 6-phosphate, lactate and GTP, while decreased the level of pyruvate. Bioinformatic analysis revealed that MV recovered multiple metabolism-related processes including gluconeogenesis, glycolysis and glucose metabolic process. Further real-time quantitative PCR analysis showed that MV upregulated the expression of lactate dehydrogenase A (*Ldha*), hexokinase 2 (*Hk2*) and pyruvate kinase M2 (*Pkm2*). Western blotting and immunohistochemistry analysis showed that MV restored the expression of lactate dehydrogenase A (*Ldha*), hexokinase 2 (*Hk2*) and pyruvate kinase M2 (*Pkm2*). Collectively, these findings indicated that MV could effectively improve the ovarian microenvironment by upregulating the expression of LDHA, HK2 and PKM2 in granulosa cells and enhancing lactate and energy production, which may contribute to follicle development and ovulation of young-adult PCOS rats.

## Introduction

Polycystic ovary syndrome (PCOS) is a complex endocrine-metabolic disorder characterized by hyperandrogenemia, ovulatory dysfunction, polycystic ovaries, and is often accompanied by insulin resistance (1, 2), abnormal glucose and lipid metabolism (3). Depending on different diagnostic criteria, the prevalence of PCOS ranges from 8 to 13% (4). Epidemiological surveys suggest that PCOS is the leading cause of infertility in women (5). However, the underlying pathogenic mechanisms of PCOS are unclear, and there are no general strategies or drugs available for the treatment of patients with PCOS (6). Recently, PCOS rats induced with letrozole and high-fat diet (HFD) showed a highly similar phenotype to that of human PCOS, providing an extremely suitable model for the study of PCOS (7). Follicular dysplasia is an important clinical characteristic of patients with PCOS (8). During follicular development, granulosa cells (GCs) play indispensable roles in oocyte differentiation and energy production, which causally affects steroidogenesis, ovulation, folliculogenesis, atresia and luteinization (9). Oocyte presents a low glycolytic rate and mainly relies on the cumulus cells to convert glucose into substrates such as pyruvate and lactate, which can be readily utilized (10). The glycolytic pathway in GCs accounts for a large proportion of glucose metabolism and provides sufficient ATP and metabolites for the oocyte. Consequently, GCs are considered to be essential components of follicular development and their energy metabolism has attracted more and more attention (9, 11).

Currently, increasing attention has been paid to screen effective and applicable drug candidates from traditional Chinese medicine (12). Mogroside V (MV), extracted from *Siraitia grosvenorii* (Luo-Han-Guo, LHG), is a triterpenoid glycoside that possesses broad pharmacological properties including antioxidant, hypoglycemic action, relieving cough, reducing sputum and etc. (13). MV presents a remarkable ability to scavenge reactive oxygen species (ROS) (14). MV can also reverse the abnormal cytoskeleton, mitochondrial dysfunction and early apoptosis of oocytes to protect against the deterioration of oocyte quality *via* up-regulating the expression of sirtuin 1 (15). In addition, MV can promote the development of porcine oocytes (16). Yan et al., demonstrated that MV protected porcine oocytes from lipopolysaccharide-induced meiotic defects (17). Recent studies also found that MV could alleviates oocyte meiotic defects and quality deterioration in benzo(a)pyrene-exposed mice (18).

To our best knowledge, the effects of MV on energy metabolism during follicular development and its underlying mechanisms remain unclear. Therefore, the study aims to evaluate the protective roles and possible underlying mechanisms of MV on ovarian dysfunction of a young-adult PCOS rat model induced by letrozole and HFD. The present study may provide a potential strategy for PCOS therapy.

## Materials and Methods

### Chemical and Reagents

Letrozole was purchased from Heng Rui Pharmaceutical Company (190605KG, Lianyungang, Jiangsu, China). Mogroside V (50.42% mogroside V), prepared from fresh fruit of *Siraitia grosvenorii*, was a generous gift from Professor Xingwei Liang, who purchased from Guilin Layn Natural Ingredients Corp. (MOV04-18022504, Guilin, Guangxi, China). Carboxymethylcellulose sodium (CMC) was purchased from Henan Wan Bang Industrial Co., Ltd. (Henan, China). TransScript^®^ One-Step gDNA Removal and cDNA Synthesis SuperMix Kit (AT311-02) were purchased from TransGen Biotech (Beijing, China). 2 × Universal SYBR Green Fast qPCR Mix kit (RK21203) was purchased from ABclonal Technology Co., Ltd. (Wuhan, China). LDHA (C28H7) rabbit mAb (#3558), hexokinase II (HK2) rabbit mAb (#2867) and PKM2 (D78A4) XP^®^ mAb (#4053) were purchased from Cell Signaling Technology, Inc. (Danvers, Massachusetts, USA). β-tubulin (No: 66240-1-Ig), horseradish peroxidase-conjugated goat anti-rabbit IgG (H+L) (SA00001-2) and biotin-conjugated affinipure goat anti- rabbit IgG (H+L) (SA00004-2) were purchased from Protein Tech Group Inc. (Chicago, USA). Wright’s–Giemsa Stain solution (G1020) and 20 × Metal Enhanced DAB Substrate Kit (DA1015) were purchased from Beijing Solarbio Science & Technology Co., Ltd (Beijing, China). BCA Protein Assay Kit (CW0014S), eECL Western Blot Kit (CW0049M) and SDS-PAGE Gel Kit (CW0022S) were purchased from Beijing ComWin Biotech Co., Ltd. (Beijing, China). Trizol reagent (15596026 and 15596018) was purchased from Thermo Fisher Scientific (Waltham, USA). Key resources of the study were also summarized in **Supplemental Tables S1, S2**. All primers were designed and synthesized by Shanghai Sangon Biotechnology Co., Ltd. (Shanghai, China).

### Animals and Induction of PCOS

Female Sprague Dawley (SD) rats (n = 30, 5 weeks old, 173.66 ± 7.73 g) were purchased from the Laboratory Animal Center of the University of South China (Hengyang, China). All animals were kept in standard environmental conditions (25°C, 12 h light/dark cycle) with free access to standard food pellets and water. After adaptive feeding for one week, the animals were randomly divided into the control group (n = 10) and the PCOS group (n = 20). The rats from the control group were fed with standard food pellets; the rats from PCOS group were orally administrated with letrozole (1 mg/kg/day, dissolved in 1% (w/w) CMC) (7) and fed with a high-fat diet (HFD, consisting of 61.5% standard food, 12% lard, 5% sucrose, 5% milk powder, 5% peanut, 10% egg, 1% sesame oil, 0.5% salt) from 1^st^ to 30^th^ days. From 21^st^ to 30^th^ day, the estrous cycle was identified by vaginal smear. According to the estrous cycle, the female rats successively stayed in metestrus and diestrus stages were preliminarily considered as PCOS models. Then, the young-adult PCOS rats were further divided into the PCOS group and the PCOS + MV group (n = 10 for each group). Rats in the PCOS + MV group were administrated through an oral gavage with mogroside V (MV, 600 mg/kg/day, dissolved in 0.9% NaCl) from 31^st^ to 60^th^ days, and vaginal cytology was performed from 51^st^ to 60^th^ days. At the end of the treatment period, all rats were anesthetized with an intraperitoneal injection of 10% chloral hydrate (0.8 ml/100 g) before sacrifice. All animal experiments were approved by the Animal Ethics Committee of University of South China (No. SYXK2020-0002).

### Vaginal Cytology

Vaginal smears were performed for 10 consecutive days at 13:00 from the 21^st^ day of modelling and initiation of MV administration, respectively. Briefly, the vagina was flushed with 25-30 μl saline (0.9% NaCl) for 2~3 times. The vaginal fluid (10-20 μl) was sampled onto the glass slides and air dried at room temperature. Then, the slides were stained with Wright’s–Giemsa Stain solution (G1020, Solarbio, Beijing, China) following the manufacturer’s protocol. Finally, the estrous cycle including proestrus, estrous, metestrus and diestrus phases and changes of vaginal epithelial cells were evaluated according to the predominant cell type (19, 20) under a light microscope (BX43, OLYMPUS, PA, USA).

### Serum Collection and Measurement of Hormone Levels

Following 12 h of fasting, blood collected from the tail vein was used to measure fasting blood glucose (FBG) with a Roche blood glucose meter (Roche diagnostic GmbH, Germany). All rats were weighed and anesthetized with 20% urethane, and the whole blood sample was collected by abdominal aortic puncture immediately. The serum was further isolated by centrifuging (2000 g) for 10 min at 4 °C. Finally, the levels of serum luteinizing hormone (LH), follicle-stimulating hormone (FSH), total testosterone and insulin were determined with radioimmunoassay (Beijing North Institute of Biotechnology Co., Ltd., Beijing, China). In brief, the serum samples (FSH: 200 μL, LH: 200 μL, testosterone: 50 μl, Insulin: 100 μL) were incubated with corresponding labeled antibodies (FSH and LH: 4 °C for 20 h, testosterone: 37 °C for 1 h, insulin: 37 °C for 2 h). Then, an immune separation agent was added to remove the uncombined antigens by mixing and centrifuging at 3800 rpm for 15 mins. After that, the radioactivity of the compound was detected to measure the corresponding hormone levels. Formula (FBG × insulin level)/22.5 was used to detect homeostasis model assessment of insulin resistance (HOMA-IR) (21).

### Morphological Analysis of Ovarian Tissues

The left ovaries of rats from each group were fixed in 4% paraformaldehyde and embedded in paraffin after serial dehydration, then were sequentially sectioned with a thickness of 6 μm and stained with hematoxylin and eosin (H&E). The morphological changes of the ovarian tissues were checked and photographed using a light microscope (BX 43; Olympus, PA, USA). In addition, the number of corpora lutea was counted in one representative section taken from the middle of the ovary under 10 × or 20 × magnification according to previous studies (22, 23).

### Targeted Energy Metabolomics With Liquid Chromatography/Tandem Mass Spectrometry (LC-MS/MS)

The right ovaries of rats from each group were sent to Shanghai Applied Protein Technology Co., Ltd. (Shanghai, China) to analyze the targeted energy metabolomics *via* LC-MS/MS. In brief, 100 mg ovarian tissues, 200 μl of ultra-pure water and 800 μl of methanol/acetonitrile (1:1, v/v) were first added into the Eppendorf tubes. Then, the samples were grounded by ultrasonic homogenizers, incubated at 20 °C for 60 min, and centrifuged at 13, 000 rpm for 15 min at 4 °C. The supernatants were harvested and dried with nitrogen, and the lyophilized powder was stored at -80 °C. The lyophilized samples were reconstituted by dissolving with 100 μl solvent mixture containing water/acetonitrile (1:1, v/v). The samples were vortexed for 1 min and centrifuged at 14, 000 rpm for 15 min at 4 °C. Finally, the supernatants were collected for LC-MS/MS analysis. A hierarchical clustering heatmap was produced to visualize the metabolites expression level using the OmicStudio tools. (https://www.omicstudio.cn/tool/4).

### Transcriptome Assay

RNA-seq library construction and RNA sequencing of ovaries (n = 4 rats per group) were performed by the Novogene Corporation (Beijing, China). Total RNA (3 μg) was used as the initial RNA for database construction. The NEBNext^®^ Ultra TM RNA Library Prep Kit (Illumina, USA) was employed to build the library. The Illumina platform was employed to sequence these libraries using a 150 bp paired-end strategy. Reads with adapter, reads with N (N means the base information cannot be determined) and low-quality reads (reads with Qphred ≤ 20 bases accounting for more than 50% of the read length) were mainly removed and filtered from the original data. The construction of the index of the reference genome and the comparison of clean reads at the mating end with the reference genome were both performed with HISA T2 v2.0.5.

### Abundance Estimation and Differential Expression Analysis for Genes

Feature Counts were used to calculate the readings mapped to each gene (24). The fragments per kilobase of transcript per million fragments mapped (FPKM) was then calculated based on the length of the gene and the readings mapped to that gene were calculated. Differential expression analysis of two conditions/groups (three biological replicates per condition) was performed using the DESeq2 R package (1.16.1). The resulting *P*-values were adjusted using Benjamini and Hochberg’s approach to control the false discovery rate (25). Genes with an adjusted *P*-value (*P*adj) < 0.05 were assigned as differentially expressed genes (DEGs).

### Gene Ontology (GO) and KEGG Enrichment Analysis of DEGs

GO enrichment analysis of DEGs was implemented by the cluster-Profiler R package. GO terms with a corrected *P*-value less than 0.05 were considered to be significantly enriched by DEGs. ClusterProfiler R package was used to test the statistical enrichment of DEGs in KEGG pathways (http://www.genome.jp/kegg/).

### Real-Time Quantitative PCR (RT-qPCR) Analysis

Total mRNA was extracted from ovarian tissues with Trizol reagent according to the manufacturer’s procedure. RNA (2 μg) was reversely transcribed into cDNA by using TransScript^®^ One-Step gDNA Removal and cDNA Synthesis SuperMix Kit. RT-qPCR was performed in 10 μl volume using 2 × Universal SYBR Green Fast qPCR Mix kit and Applied Biosystems QuantStudio 3 (Thermo Fisher Scientific). *Gapdh* was used as the reference and gene expression levels were calculated using the 2^-ΔΔCt^ method. The mRNA expression levels were calculated as relative expression to *Gapdh*. All the primers were summarized in **Table 1**, and the Ct values were summarized in **Supplemental Table S3**.

**Table 1:** Primer sequences used for the qRT-PCR analysis.

Gene Name	Sequence (5’-3’)	Annealing Temperature	Product (bp)	GeneBank No	Ct of Control group	Ct of PCOS group	Ct of PCOS-MV group
HK2	F: GTGGTGAATGACACAGTTGG	55 °C	120	NM_012735.2	28.799±1.32	30.157±1.429	26.709±1.150
	R: CACATTACGCATCTCTTCCA						
PKM2	F: ACATCCTGTGGCTGGACTAT	55 °C	130	NM_053297.2	24.042±2.577	24.758±1.619	21.301±1.888
	R: TCCACTTCTGTCACCAGGTA						
LDHA	F: GGTTGACAGTGCATACGAAG	55 °C	106	NM_017025.2	25.336±2.175	26.216±1.262	23.516±2.277
	R: CCGCCTAAGGTTCTTCATTA						
GAPDH	F: CCTCAAGATTGTCAGCAATG	55 °C	134	NM_017008.4	20.817±2.799	20.417±1.923	18.065±2.293
	R: CAGTCTTCTGAGTGGCAGTG						

### Immunohistochemistry

Immunohistochemical analysis was conducted in accordance with a previous study with minor modification (21). Immunohistochemistry was performed on formalin-fixed and paraffin-embedded specimens with primary antibodies including polyclonal LDHA antibodies (1:300 dilution), HK2 antibodies (1:400 dilution) and PKM2 antibodies (1:500 dilution), respectively. The immunoreactive signals of proteins were visualized with 20 × Metal Enhanced DAB Substrate Kit. Finally, sections were observed and photographed with an Olympus DP70 digital camera mounted on a Leica DMR microscope.

### Western Blotting

Western blotting (WB) assay was performed as described previously (26). Briefly, the protein concentration was detected by a BCA Protein Assay Kit. Briefly, 40 μg protein samples were separated with 10% SDS-PAGE and transferred to polyvinylidene fluoride membranes (Meck Millipore, Merck & Co., Inc., NJ, USA). Then, transferred membranes were blocked by 5% skim milk for 1 h at room temperature. The primary rabbit monoclonal antibodies including LDHA (1:1000 dilution), HK2 (1:1000 dilution), PKM2 (1:1000 dilution), and β-tubulin (1:3000 dilution) were respectively incubated overnight at 4°C, and the incubation of a horseradish peroxidase-conjugated goat anti-rabbit IgG (1:5000 dilution) was performed for 1 h at room temperature. β-tubulin was detected as a loading control. Finally, eECL was added, and the Tanon-5500 Chemiluminescence Imaging System was used to detect the chemiluminescence of protein bands. Finally, blotting images were quantified using Image J analysis software (JAVA image processing program, NIH, Bethesda, USA).

### Statistical Analysis

Bioinformatics analysis was performed with the OmicStudio tools (https://www.omicstudio.cn/tool). For all the data results, intra-assay coefficient of variation was < 10%, and inter-assay coefficient of variation was < 15%. All data were shown as mean ± standard error of the mean (SEM) unless otherwise stated. Differences between multiple groups were analyzed by one-way analysis of variance (ANOVA) followed by Bonferroni’s multiple comparison test. All statistical analyses were performed using GraphPad Prism 9 (GraphPad Prism, USA). A *p*-value less than 0.05 was considered statistically significant.

## Results

### Effects of MV on Body Weight and the Estrous Cycle of Young-Adult PCOS Rats

**Figure 1:**
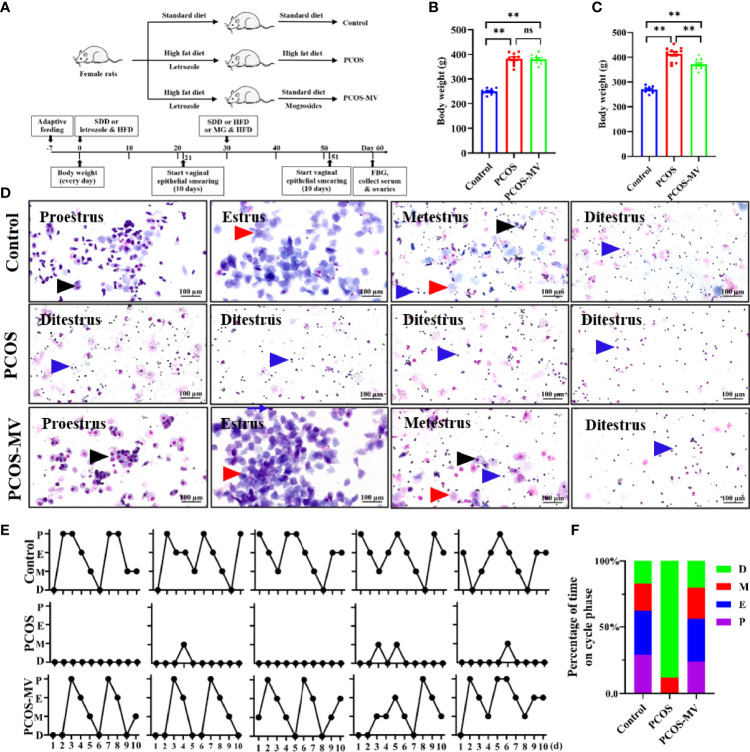
Effects of MV administration on body weight and vaginal cytology of PCOS rats. **(A)** Schematic diagram showing the experimental treatment of PCOS and PCOS-MV rats. **(B)** Body weight of rats after modeling (n = 10, day 30). **(C)** Body weight of rats after MV treatment (n = 10, day 60). **(D)** Cytological assessment of vaginal smears (n = 10, day 51-60). **(E)** Representative estrous cycles. **(F)** Quantitative analysis about the percentage of time on different phase of estrous cycles (n = 10 per group). Significant differences between groups were indicated as **P < 0.01. not significant difference (*P* > 0.05). MV, mogroside V.

To identify the effects of MV, young-adult PCOS rat model was established, and the experimental design was shown in **Figure 1A**. There was a remarkable increase in body weight of the rats after the treatment of HFD and letrozole (**Figure 1B** and **Table 2**), and this increase was significantly reduced after MV administration (**Figure 1C** and **Table 2**). Estrous disorder is one of the main characteristics of PCOS rats (27, 28), so the effects of MV on the estrous cycle of young-adult PCOS rats were conducted. In the control group, the rats had a regular estrous cycle (4-5 days) including proestrus, estrous, metestrus and diestrus phases sequentially (**Figures 1D, E**), which is consistent with previous studies (19, 20, 29). In the proestrus phase, there were mainly small and round nucleated epithelial cells with greyish red nucleus and greyish cytoplasm (black arrows point), which were relatively uniform in appearance and size. Estrous phase was predominantly characterized with flat and anucleated keratinized epithelial cells showing as blue or purple-blue stacks or layers (red arrows point). However, in the metestrus phase, neutrophils and nuclear epithelial cells were the predominant compositions, and anucleated keratinized epithelial cells were occasionally observed. In the diestrus phase, there were almost full of neutrophils (**Figure 1D**, blue arrows point). Compared with the controls group, young-adult PCOS rats showed disrupted estrous cycles with prolonged diestrus but shortened proestrus and estrous phases (**Figure 1F**). Intriguingly, the irregular estrous cycle was gradually returned to be normal after MV administration (**Figures 1D–F**).

**Table 2:** Measurements of body weight, ovary weight and hormone concentrations in Control, PCOS and PCOS-MV groups.

Parameters	Control	PCOS	PCOS-MV
	n = 10	n = 10	n = 10
Body weight (g) (After modeling)	249.3 ± 3.703^**^	382.4±7.488	380.3 ± 5.853
Body weight (g) (After MV treatment)	268.9 ± 3.944^**^	413.4 ± 9.321	371.5 ± 6.782^**^
ovary (mg)	94.16 ± 3.249^**^	139.1 ± 5.306	113.3 ± 6.055^**^
FBG	8.690 ± 0.738	9.39 ± 0.429	9.710 ± 0.261
FSH (mIU/ml)	1.464 ± 0.213	2.271 ± 0.263	1.941 ± 0.215
LH (mIU/ml)	4.771 ± 0.282	4.570 ± 0.412	3.390 ± 0.500
T (ng/ml)	0.116 ± 0.013^**^	2.587 ± 0.517	0.078 ± 0.020^**^
Insulin (pg/ml)	39.92 ± 3.412^**^	73.36 ± 5.806	68.43 ± 9.541
LH/FSH	3.925 ± 0.689	2.323 ± 0.339	1.793 ± 0.209
HOMO-IR	15.41 ± 1.863^**^	31.33 ± 3.686	29.51 ± 4.25

PCOS, polycystic ovary syndrome; MV, mogroside V; FSH, follicle-stimulating hormone; T, testosterone; LH, luteinizing hormone; LH/FSH, LH/FSH ratio; HOMO-IR, Homeostatic model assessment for insulin resistance. Data presented as mean ± SEM. ^∗∗^P < 0.01, Control vs PCOS and PCOS-MV vs PCOS groups.

### Effects of MV on Hormone Levels, Ovary Weight and Ovary Morphology

**Figure 2:**
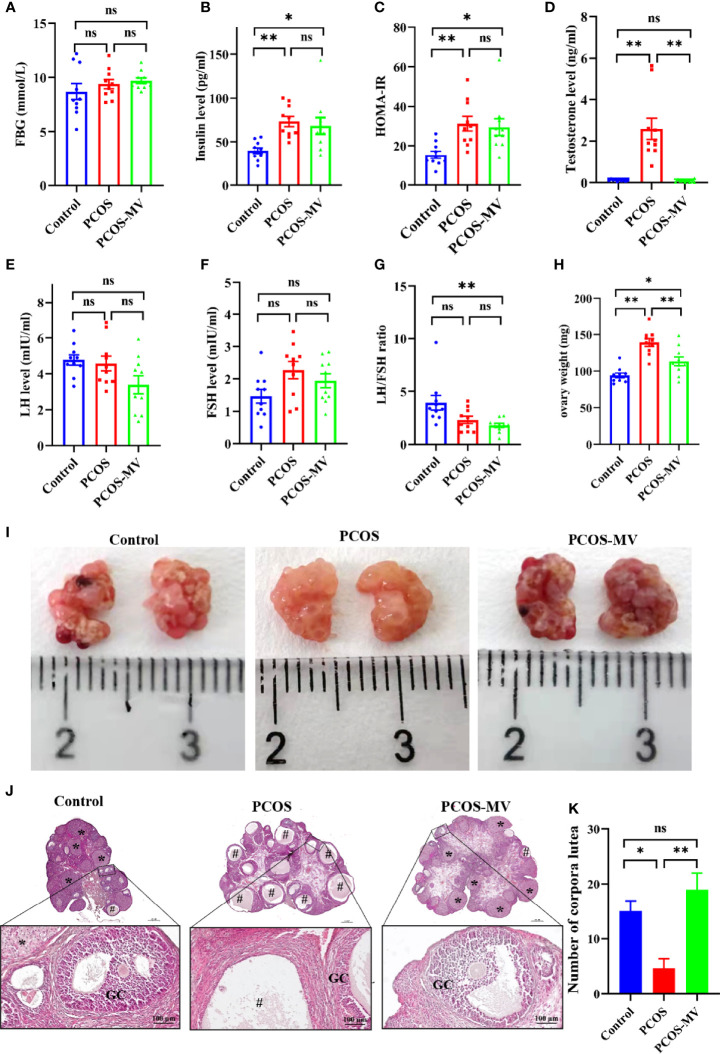
Effects of MV administration on hormone level, ovary weight and ovary morphology. **(A)** FBG (n = 10 rats per group). **(B)** Insulin (n = 10). **(C)** HOMA-IR (n = 10). **(D-F)** Serum levels of sex hormone indicators including testosterone (T), luteinizing hormone (LH) and follicle-stimulating hormone (FSH) (n = 10). **(G)** LH/FSH ratio. **(H)** Effects of MV treatment on the ovary weight of PCOS rats (n = 10). **(I)** Appearance of representative ovaries. Dark red regions show the corpora lutea. **(J)** Histological structure of ovarian tissues (n = 6 rats per group, scale bar in upper is 500 μm). The lower panel show the higher magnification of the box area in the upper panel respectively. **(K)** Quantification of the number of corpora lutea (n = 6). “#” indicated the cystic follicles, “*” indicated the corpus luteum. Significant differences between groups were indicated as *P < 0.05, **P < 0.01. GC, granulosa cell. ns, no significance.

To reveal the underpinning mechanism of protective effects of MV administration on dysfunctional estrous cycle, we further evaluated the effects of MV treatment on the hormone levels, ovary weight and ovary morphology of young-adult PCOS rats. There were no significant differences in FBG among control, PCOS and PCOS-MV groups (**Figure 2A** and **Table 2**). Compared with the control group, though obvious increase was found in the serum level of insulin, HOMA-IR and testosterone of PCOS group, MV treatment only overtly reduced the level of testosterone in the PCOS-MV group, even there was downtrend without statistical significance found in insulin level and HOMA-IR (**Figures 2B-D** and **Table 2**). In addition, there were no statistical differences in the serum levels of LH, FSH and LH/FSH between control *vs* PCOS and PCOS *vs* PCOS-MV groups (**Figures 2E–G**). The ovary weight of PCOS group significantly increased, while that of PCOS-MV group apparently decreased (**Figure 2H**). Moreover, to check the improvement of MV on the folliculogenesis and ovulation of young-adult PCOS rats, we analyzed histological morphology of ovary and counted the number of corpora lutea. Compared with the control group, multiple follicles with cystic expansion presented vacuolated and disorganized structure and only a few corpora lutea were found in the PCOS group. However, follicles at different stages and obviously increased numbers of corpus luteum were observed in the control and PCOS-MV groups (**Figures 2I–K**). Interestingly, the MV administration recovered the thickness of granulosa cell layers (**Figures 2I–K**).

### Effects of MV on Energy Metabolism of the Ovary

**Figure 3:**
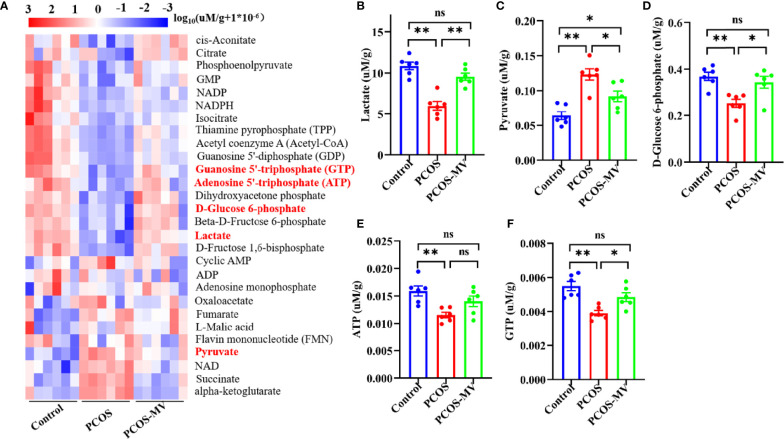
Targeted energy metabolomics analysis of ovarian tissues. **(A)** Cluster analysis of energy metabolites among control, PCOS and PCOS-MV groups (n = 6 rats per group). **(B)** Lactate. **(C)** Pyruvate. **(D)** D-Glucose 6-phosphate. **(E)** ATP. **(F)** GTP. Significant differences between groups were respectively indicated as*P < 0.05 and **P < 0.01.

Given the key roles of glycolysis in the development and mature of follicles (10), we mainly focused on the main metabolites of glycolysis including D-Glucose 6-phosphate, pyruvate, lactate, ATP and GTP (**Figure 3A**, red fonts). Compared with the control group, the production of D-Glucose 6-phosphate, lactate, ATP and GTP in the PCOS group was significantly down-regulated excepting an obvious up-regulation in the production of pyruvate (**Figures 3A–F**). MV administration markedly increased the levels of D-Glucose 6-phosphate, lactate, ATP and GTP, and inversely decreased the pyruvate level (**Figures 3A–F**).

### Effects of MV on the Transcriptome of Ovary

**Figure 4:**
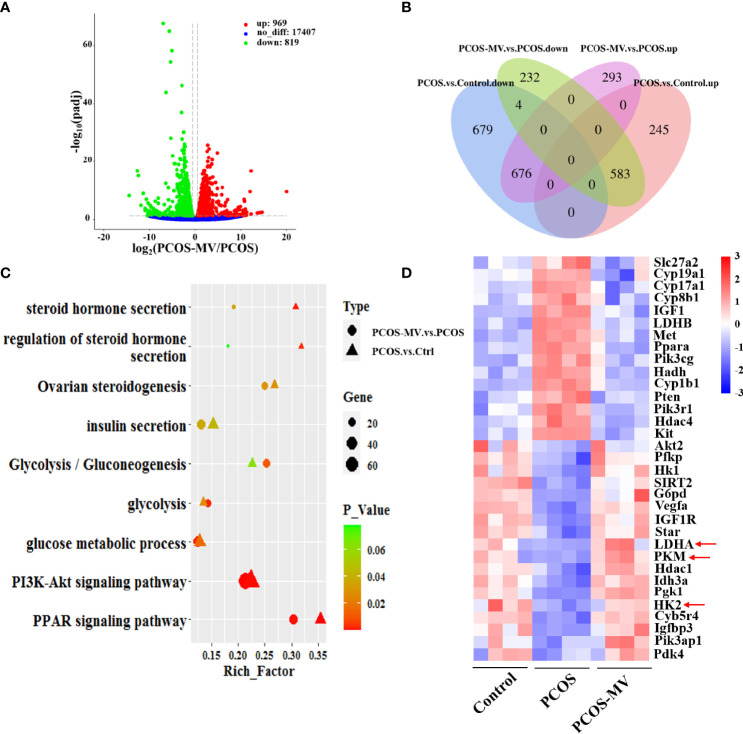
Transcriptome analysis of ovarian tissues. **(A)** Volcano plot of genes in the comparison of PCOS-MV *vs* PCOS groups (n = 4). **(B)** Comparison of common DEGs between the PCOS *vs* Control and PCOS-MV *vs* PCOS. **(C)** Comparison of functional enrichment of DEGs between the PCOS-*vs*-Control and PCOS-MV *vs* PCOS. **(D)** Heatmap of DEGs related to energy and hormone metabolism. up: upregulated. down: downregulated.

To further explore the molecular mechanisms underlying the recovery of MV administration on the development and energy metabolism of ovaries, we performed transcriptome sequencing (RNA-seq) of ovarian tissues. There were respective 828 upregulated and 1359 downregulated genes in the PCOS group when compared with the control group (26). Compared to the PCOS group, 969 and 819 genes were respectively upregulated and downregulated in rats from the PCOS-MV group (**Figure 4A**), among which 583 of the 828 genes upregulated genes and 676 of 1359 downregulated in the PCOS group were significantly reversed after MV administration (**Figure 4B**). These reversed genes are considered as MV-responsive genes (**Figure 4B**). In addition, the DEGs were involved in metabolic pathways, such as glycolysis, PI3K-Akt signaling pathway and peroxisome proliferator-activated receptor signaling pathway (**Figure 4C**). Notably, MV administration restored these metabolism-related biological processes, which were dysregulated in young-adult PCOS rats (**Figure 4D**). The expression levels of several genes related to glycolysis such as LDHA, HK2 and PKM exhibited a reversed phase between the PCOS and PCOS-MV groups (**Figure 4D**). These results led to the speculation that MV may improve the glycolysis in the ovaries of young-adult PCOS rats.

### MV Reversed the Decrease of LDHA, HK2 and PKM2 in Young-Adult PCOS Rats

**Figure 5:**
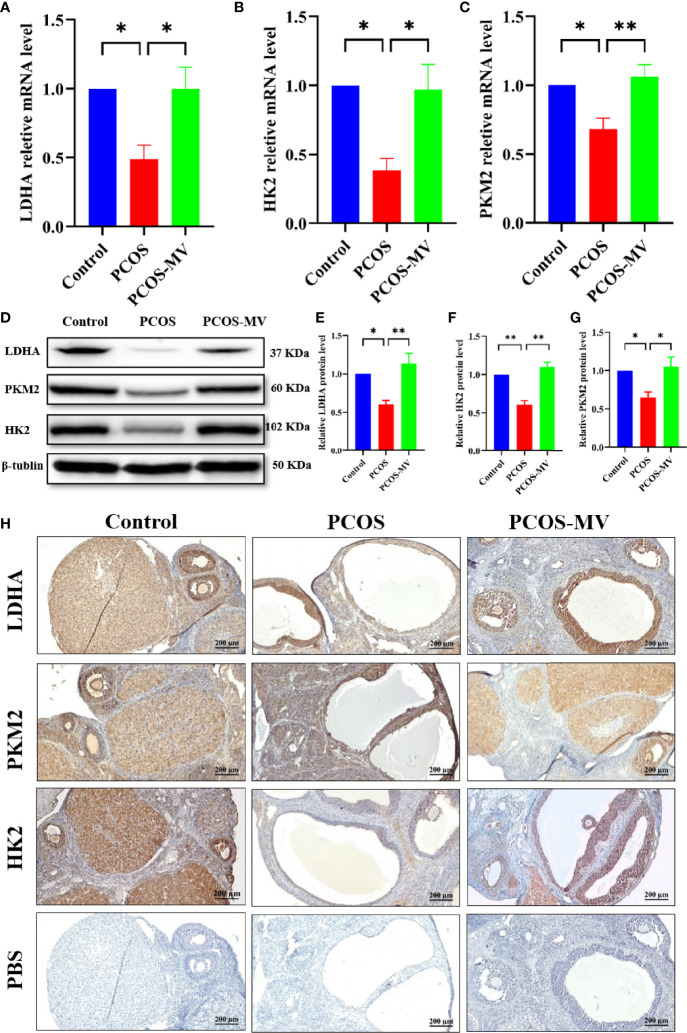
Expression of LDHA, PKM2 and HK2 in ovarian tissues. **(A)** Relative *Ldha* mRNA level of ovaries (n = 4 rats per group). **(B)** Relative *Pkm2* mRNA level of ovaries (n = 4 rats per group). **(C)** Relative *Hk2* mRNA level of ovaries (n = 4 rats per group). **(D)** Western blotting assays of LDHA, PKM2 and HK2 in ovarian tissues, with β-tubulin served as a loading control (n = 4 rats per group). **(E)** Relative LDHA protein level of ovaries (n = 4 rats per group). **(F)** Relative PKM2 protein level of ovaries (n = 4 rats per group). **(G)** Relative HK2 protein level of ovaries (n = 4 rats per group). **(H)** Immunohistochemistry assays of LDHA, PKM2 and HK2 in ovarian tissues (n = 4 rats per group), the lowest panel shows the negative control. Data were presented as mean ± SEM. Significant differences were respectively presented as *P < 0.05 and **P < 0.01.

ATP production originally relies on the levels of glycolytic rate-limiting enzymes, which play indispensable roles in controlling glycolysis rate (30). LDHA, HK2 and PKM2 are key rate-limiting enzymes of glycolysis (31–33), which regulates the energy supply of oocytes and GCs (34). Given the above-mentioned transcriptome profiling that shows the reversed expression profile of LDHA, HK2 and PKM between the PCOS and PCOS-MV groups (**Figure 4D**), we further detected the expression levels of the three glycolytic rate-limiting enzymes. Compared with the control group, the mRNA expression levels of *Ldha*, *Pkm2* and *Hk2* were significantly down-regulated, MV administration obviously increased the mRNA expression of *Ldha*, *Pkm2* and *Hk2* (**Figures 5A–C**). WB analysis presented the expression of LDHA, HK2 and PKM2 were significantly down-regulated in the ovarian tissues of young-adult PCOS rats, which was significantly up-regulated after MV administration (**Figures 5D–G**). Immunohistochemistry (IHC) analysis further showed that LDHA, HK2 and PKM2 markedly decreased in the PCOS group as compared with the control group and recovered in the PCOS-MV group (**Figure 5H**).

## Discussion

In this study, we established a young-adult PCOS rat model exhibiting characteristics of obesity, hyperandrogenism and polycystic ovaries through the oral administration of letrozole and HFD and examined the roles of MV on the development of follicles and ovulation by intragastric administration of MV. MV is reported to promote *in vitro* maturation of oocytes (16). This study for the first time proved the therapeutic effects of MV on follicle development and ovulation in young-adult rats (about 10 weeks old) with PCOS *in vivo* by upregulating the expression of LDHA, HK2 and PKM2 to improve the energy supply of follicle development.

**Figure 6:**
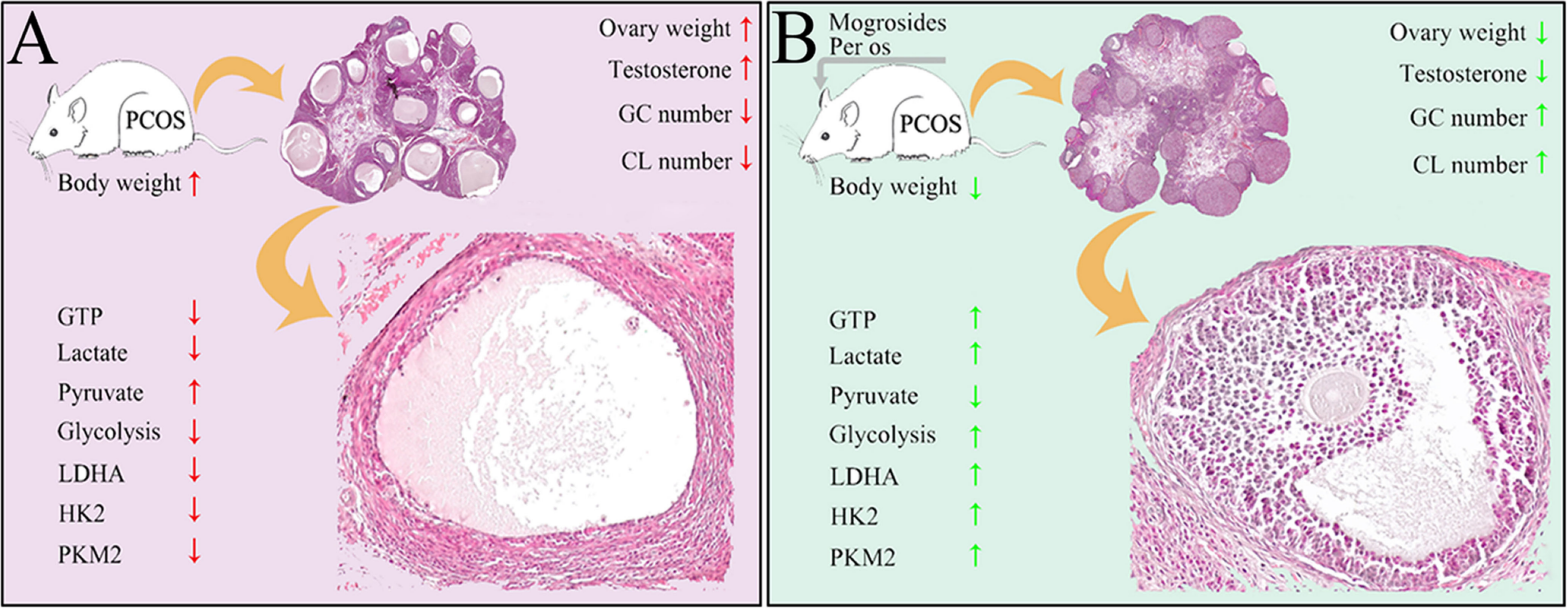
Schematic illustrating the protective roles of MV on follicular development and ovulation of PCOS rats possibly through regulating the glycolysis pathway. **(A)** Ovarian damages caused by the treatment of high-fat diet and letrozole in PCOS rats. Red arrows present bad effects (with significant difference to control) on ovarian function. **(B)** The restoring functions of MV to ovarian damages of PCOS rats. Green arrows mean obvious improvement after MV treatment. GC, granulosa cell. CL, corpus luteum.

According to previous results, PCOS severely impaired female reproduction due to anovulation. In the dehydroepiandrosterone (DHEA)- or letrozole-induced PCOS rats/mice, they all exhibited increased body weight and ovary weight, disrupted estrous cycle, reduction of corpus luteum, decreased thickness of granular layers and increased apoptosis of GCs (35–38). In this study, letrozole- and HFD-induced young-adult PCOS rats showed almost identical reproductive dysfunctions. Inversely, MV administration restored the abnormal estrous cycle, decreased the body weight and ovary weight, recovered the number of corpus luteum and the thickness of granular layers. These results indicated that MV could effectively improve follicular development and ovulation in young-adult PCOS rats (**Figure 6**).

Owning to the low glycolytic activity of oocytes that preferentially make use of metabolic substrates from GCs for energy homeostasis (10), the energy production of folliculogenesis predominantly relied on glycolysis of GCs (39). Therefore, dysfunction of energy metabolism in GCs could causally lead to aberrantly follicular development. Pyruvate and lactate, products of glycolysis in GCs, are two main energy substrates that play vital roles during folliculogenesis (40). Normally, the level of lactate in follicular fluid would increase with the maturity and size of the developing follicle (41). Studies have shown that there were disorders of glycolysis and pyruvate metabolism in follicular fluids of PCOS patients (42). Herein, the production of D-Glucose 6-phosphate, lactate, ATP and GTP in young-adult PCOS rats decreased significantly, while the production of pyruvate increased markedly (**Figure 3**). These metabolic characteristics were highly similar to previous studies showing that Diane-35 combined with metformin treatment improved the ovulation in the PCOS rat model possibly *via* regulating glycolysis pathway (43). On the other hand, MV administration markedly increased the levels of D-Glucose 6-phosphate, lactate, ATP and GTP, and decreased the level of pyruvate. In addition, transcriptome analysis revealed the dysregulated metabolism-related processes including glycolysis, but MV administration restored the abnormal glycolysis. Moreover, the expression profiles of three glycolytic rate-limiting enzymes (LDHA, HK2 and PKM2) between PCOS and PCOS-MV groups further confirmed that MV could improve the disrupted glycolysis in the ovarian tissues of young-adult PCOS rats. Consistently, previous studies also showed that resveratrol upregulated LDHA, HK2, and PKM2 in the PCOS rats (26). Overall, these results indicated that MV improved ovulation potentially through promoting the glycolysis pathway, HK2 and PKM2 promoted pyruvate production, and LDHA promoted glycolysis process by catalyzing the conversion of pyruvate to lactate (**Figure 6**).

Although numerous factors are responsible for the occurrence of PCOS, androgen is thought to be the pivotal one (44–46). Hyper-androgen could induce ovarian GC pyroptotic death and follicular dysfunction in PCOS mice (47), preventing oocytes from maturation and ovulation. The levels of testosterone were all significantly increased in PCOS models induced by different drugs (35–38). In this study, obvious up-regulation of testosterone and thinness of granular layers were also found in young-adult PCOS rats induced by letrozole and HFD, and MV administration apparently reduced the level of testosterone to normal and recovered the thickness of the granular layers, showing high effectiveness of MV on the restoring of PCOS symptoms. In addition to the increase of testosterone level, there were significantly elevated levels of the LH and LH/FSH ratio in PCOS models induced by letrozole (27, 37, 43, 48). However, the levels of LH and FSH or LH/FSH ratio were found to be no statistical differences in PCOS mice or rats induced by DHEA (35, 49). Our results were considered consistent with the latter, and this could probably be due to the limited number of samples. Moreover, as a common endocrine disorder, PCOS caused metabolic dysregulation that increased the morbidity of type 2 diabetes mellitus (T2DM) (27, 50). PCOS was often associated with insulin resistance and defects in insulin secretion (51, 52). Apparent abnormality of FBG, insulin level and HOMA-IR were found in different PCOS models (27, 35, 43). Furthermore, Zhang et al., demonstrated that MV have the potential to improve the conditions of hyperglycemia and regulate the secretion of insulin in T2DM rats (53). Here, although the levels of FBG, insulin and HOMA-IR were significantly increased in young-adult PCOS rats, there was only a downward trend without statistical significance after MV administration, while underlying mechanisms need to be further studied.

The present study has several limitations. Firstly, the rats used in this study were limited to young-adult rats, while the age has a significant impact on the ovary development, thus, future studies should confirm our findings in the rats with wide ranges of ages. Secondly, the acclimation of only one week for this type of study may be not sufficient, and future studies should consider longer period for acclimation. Thirdly, chemical reagents used for anesthetizing the rats may affect hormone balance, and we should cautious when interpreting these results. Fourthly, the stage of estrous cycle was assessed for only 10 days in our study, which may not be sufficient during such dynamic period of female reproductive life, thus, future studies may extend the duration for monitoring the stage of estrous cycle.

## Conclusion

In conclusion, this study demonstrated that MV effectively improved follicular development and ovulation of young-adult PCOS rats induced by letrozole and HFD through accelerating glycolysis pathway to generate more energy, providing a potential therapeutic strategy for the therapy of female infertility caused by PCOS.

## Data Availability Statement

The datasets presented in this study can be found in online repositories. The names of the repository/repositories and accession number(s) are as follows: GEO, GSE194398.

## Ethics Statement

All animal experiments were approved by the Animal Ethics Committee of University of South China (No. SYXK2020-0002).

## Author Contributions

LHuang: investigation, formal analysis, visualization, software, and writing-original draft. AL: investigation, formal analysis, validation, and writing-review and editing. TL: investigation and writing-review and editing. LHu: investigation and formal analysis. XChen: funding acquisition and supervision. JT, XCao, GC, FC, and YW: investigation. XL: methodology, supervision, and writing-review and editing. ML: funding acquisition, methodology, writing-review and editing, and project administration. WH: visualization, writing-original draft, and writing-review and editing. All authors contributed to the article and approved the submitted version.

## Funding

This work was supported by the Scientific Research Foundation of Hunan Provincial Education Department (No. 20A434), National Natural Science Foundation of China (82101720), Doctoral research start-up funds of the University of South China (190XQD144), The Natural Science Foundation of Guangxi in China (No. 2021GXNSFBA220010). Scientific Research Elevation Project of Young Faculty from Guangxi Universities (2019KY0567); Doctoral research start-up funds of the University of South China (2014XQD15), Open Fund of State Key Laboratory of Developmental Biology of Freshwater Fish (2017KF008), Research and Innovation Program for Graduate Students of Hunan Province (CX20210959), National College Students Innovation and Entrepreneurship Training Program (S202010555067).

## Conflict of Interest

The authors declare that the research was conducted in the absence of any commercial or financial relationships that could be construed as a potential conflict of interest.

## Publisher’s Note

All claims expressed in this article are solely those of the authors and do not necessarily represent those of their affiliated organizations, or those of the publisher, the editors and the reviewers. Any product that may be evaluated in this article, or claim that may be made by its manufacturer, is not guaranteed or endorsed by the publisher.

## Abbreviations

PCOS, polycystic ovary syndrome; MV, mogroside V; HFD, high fat diet; LDHA, lactate dehydrogenase A; HK2, hexokinase 2; PKM2, pyruvate kinase M2; GCs, granulosa cells; ROS, reactive oxygen species; SD, Sprague Dawley; FBG, fasting blood glucose; INS, insulin; HOMO-IR, homeostasis model assessment-insulin resistance index; IHC, Immunohistochemistry; WB, Western blotting; DEGs, differentially expressed genes; LH, luteinizing hormone; FSH, follicle stimulating hormone; CMC, carboxymethylcellulose sodium; LC-MS/MS, liquid chromatography-tandem mass spectrometry; GO, Gene Ontology; DHEA, dehydroepiandrosterone; T2DM, type 2 diabetes mellitus; SEM: standard error of the mean; CL, corpora lutea.

## References

[1] Belenkaia LV Lazareva LM Walker W Lizneva DV Suturina LV. Criteria, Phenotypes and Prevalence of Polycystic Ovary Syndrome. Minerva Ginecol (2019) 71(3):211–23. doi: 10.23736/S0026-4784.19.04404-6 31089072

[2] Wang J Wu D Guo H Li M. Hyperandrogenemia and Insulin Resistance: The Chief Culprit of Polycystic Ovary Syndrome. Life Sci (2019) 236:116940. doi: 10.1016/j.lfs.2019.116940 31604107

[3] Akbarzadeh M Naderi T Dabbaghmanesh MH. The Glucose Metabolism Disorder and Dyslipidemia Among Girls With Different Phenotype Polycystic Ovary Syndrome. J Res Med Sci (2019) 24:72. doi: 10.4103/jrms.JRMS_804_16 31523258 PMC6734671

[4] Bozdag G Mumusoglu S Zengin D Karabulut E Yildiz BO. The Prevalence and Phenotypic Features of Polycystic Ovary Syndrome: A Systematic Review and Meta-Analysis. Hum Reprod (2016) 31(12):2841–55. doi: 10.1093/humrep/dew218 27664216

[5] Deshpande PS Gupta AS. Causes and Prevalence of Factors Causing Infertility in a Public Health Facility. J Hum Reprod Sci (2019) 12(4):287–93. doi: 10.4103/jhrs.JHRS_140_18 PMC6937760 32038077

[6] Escobar-Morreale HF. Polycystic Ovary Syndrome: Definition, Aetiology, Diagnosis and Treatment. Nat Rev Endocrinol (2018) 14(5):270–84. doi: 10.1038/nrendo.2018.24 29569621

[7] Wang MX Yin Q Xu X. A Rat Model of Polycystic Ovary Syndrome With Insulin Resistance Induced by Letrozole Combined With High Fat Diet. Med Sci Monitor Int Med J Exp Clin Res (2020) 26:e922136. doi: 10.12659/MSM.922136 PMC7268891 32448863

[8] Franks S McCarthy MI Hardy K. Development of Polycystic Ovary Syndrome: Involvement of Genetic and Environmental Factors. Int J Androl (2006) 29(1):278–85; discussion 86-90. doi: 10.1111/j.1365-2605.2005.00623.x 16390494

[9] Chronowska E. High-Throughput Analysis of Ovarian Granulosa Cell Transcriptome. BioMed Res Int (2014) 2014:213570. doi: 10.1155/2014/213570 24711992 PMC3966335

[10] Sutton-McDowall ML Gilchrist RB Thompson JG. The Pivotal Role of Glucose Metabolism in Determining Oocyte Developmental Competence. Reproduction (2010) 139(4):685–95. doi: 10.1530/REP-09-0345 20089664

[11] Dumesic DA Padmanabhan V Chazenbalk GD Abbott DH. Polycystic Ovary Syndrome as a Plausible Evolutionary Outcome of Metabolic Adaptation. Reprod Biol Endocrinol RB&E (2022) 20(1):12. doi: 10.1186/s12958-021-00878-y 35012577 PMC8744313

[12] Lin AX Chan G Hu Y Ouyang D Ung COL Shi L. Internationalization of Traditional Chinese Medicine: Current International Market, Internationalization Challenges and Prospective Suggestions. Chin Med (2018) 13:9. doi: 10.1186/s13020-018-0167-z 29449877 PMC5807832

[13] Li C Lin LM Sui F Wang ZM Huo HR Dai L. Chemistry and Pharmacology of Siraitia Grosvenorii: A Review. Chin J Nat Med (2014) 12(2):89–102. doi: 10.1016/S1875-5364(14)60015-7 24636058

[14] Qi XY Chen WJ Zhang LQ Xie BJ. Mogrosides Extract From Siraitia Grosvenori Scavenges Free Radicals *In Vitro* and Lowers Oxidative Stress, Serum Glucose, and Lipid Levels in Alloxan-Induced Diabetic Mice. Nutr Res (2008) 28(4):278–84. doi: 10.1016/j.nutres.2008.02.008 19083420

[15] Nie J Sui L Zhang H Zhang H Yan K Yang X. Mogroside V Protects Porcine Oocytes From *In Vitro* Ageing by Reducing Oxidative Stress Through SIRT1 Upregulation. Aging (Albany NY) (2019) 11(19):8362–73. doi: 10.18632/aging.102324 PMC6814602 31586990

[16] Nie J Yan K Sui L Zhang H Zhang H Yang X. Mogroside V Improves Porcine Oocyte *In Vitro* Maturation and Subsequent Embryonic Development. Theriogenology (2020) 141:35–40. doi: 10.1016/j.theriogenology.2019.09.010 31518726

[17] Yan K Cui K Nie J Zhang H Sui L Zhang H. Mogroside V Protects Porcine Oocytes From Lipopolysaccharide-Induced Meiotic Defects. Front Cell Dev Biol (2021) 9:639691. doi: 10.3389/fcell.2021.639691 33763421 PMC7982822

[18] Sui L Yan K Zhang H Nie J Yang X Xu CL. Mogroside V Alleviates Oocyte Meiotic Defects and Quality Deterioration in Benzo(a)pyrene-Exposed Mice. Front Pharmacol (2021) 12:722779. doi: 10.3389/fphar.2021.722779 34512349 PMC8428525

[19] Marcondes FK Bianchi FJ Tanno AP. Determination of the Estrous Cycle Phases of Rats: Some Helpful Considerations. Braz J Biol (2002) 62(4a):609–14. doi: 10.1590/S1519-69842002000400008 12659010

[20] Cora MC Kooistra L Travlos G. Vaginal Cytology of the Laboratory Rat and Mouse: Review and Criteria for the Staging of the Estrous Cycle Using Stained Vaginal Smears. Toxicol Pathol (2015) 43(6):776–93. doi: 10.1177/0192623315570339 PMC11504324 25739587

[21] Lei X Huo P Wang Y Xie Y Shi Q Tu H. Lycium Barbarum Polysaccharides Improve Testicular Spermatogenic Function in Streptozotocin-Induced Diabetic Rats. Front Endocrinol (Lausanne) (2020) 11:164. doi: 10.3389/fendo.2020.00164 32362869 PMC7181356

[22] Donato J Jr. Silva RJ Sita LV Lee S Lee C Lacchini S. The Ventral Premammillary Nucleus Links Fasting-Induced Changes in Leptin Levels and Coordinated Luteinizing Hormone Secretion. J Neurosci Off J Soc Neurosci (2009) 29(16):5240–50. doi: 10.1523/JNEUROSCI.0405-09.2009 PMC2696192 19386920

[23] Simavli S Thompson IR Maguire CA Gill JC Carroll RS Wolfe A. Substance P Regulates Puberty Onset and Fertility in the Female Mouse. Endocrinology (2015) 156(6):2313–22. doi: 10.1210/en.2014-2012 PMC4430622 25856429

[24] Liao Y Smyth GK Shi W. Featurecounts: An Efficient General Purpose Program for Assigning Sequence Reads to Genomic Features. Bioinformatics (2014) 30(7):923–30. doi: 10.1093/bioinformatics/btt656 24227677

[25] Benjamini Y Drai D Elmer G Kafkafi N Golani I. Controlling the False Discovery Rate in Behavior Genetics Research. Behav Brain Res (2001) 125(1-2):279–84. doi: 10.1016/S0166-4328(01)00297-2 11682119

[26] Liang A Huang L Liu H He W Lei X Li M. Resveratrol Improves Follicular Development of PCOS Rats by Regulating the Glycolytic Pathway. Mol Nutr Food Res (2021) 65(24):e2100457. doi: 10.1002/mnfr.202100457 34664388

[27] Arroyo P Ho BS Sau L Kelley ST Thackray VG. Letrozole Treatment of Pubertal Female Mice Results in Activational Effects on Reproduction, Metabolism and the Gut Microbiome. PloS One (2019) 14(9):e0223274. doi: 10.1371/journal.pone.0223274 31568518 PMC6768472

[28] Nallathambi A Bhargavan R. Regulation of Estrous Cycle by Cynodon Dactylon in Letrozole Induced Polycystic Ovarian Syndrome in Wistars Albino Rats. Anat Cell Biol (2019) 52(4):511–17. doi: 10.5115/acb.19.114 PMC6952683 31949991

[29] Goldman JM Murr AS Cooper RL. The Rodent Estrous Cycle: Characterization of Vaginal Cytology and its Utility in Toxicological Studies. Birth Defects Res B Dev Reprod Toxicol (2007) 80(2):84–97. doi: 10.1002/bdrb.20106 17342777

[30] Yan L Raj P Yao W Ying H. Glucose Metabolism in Pancreatic Cancer. Cancers (Basel) (2019) 11(10):1460. doi: 10.3390/cancers11101460 PMC6826406 31569510

[31] Feng Y Xiong Y Qiao T Li X Jia L Han Y. Lactate Dehydrogenase A: A Key Player in Carcinogenesis and Potential Target in Cancer Therapy. Cancer Med (2018) 7(12):6124–36. doi: 10.1002/cam4.1820 PMC6308051 30403008

[32] Feng J Li J Wu L Yu Q Ji J Wu J. Emerging Roles and the Regulation of Aerobic Glycolysis in Hepatocellular Carcinoma. J Exp Clin Cancer Res (2020) 39(1):126. doi: 10.1186/s13046-020-01629-4 32631382 PMC7336654

[33] Zhu S Guo Y Zhang X Liu H Yin M Chen X. Pyruvate Kinase M2 (PKM2) in Cancer and Cancer Therapeutics. Cancer Lett (2020) (503):240–48. doi: 10.1016/j.canlet.2020.11.018 33246091

[34] Wang J Wu X. The Effects of Mitochondrial Dysfunction on Energy Metabolism Switch by HIF-1alpha Signalling in Granulosa Cells of Polycystic Ovary Syndrome. Endokrynol Pol (2020) 71(2):134–45. doi: 10.5603/EP.a2020.0002 32096549

[35] Guo Y Qi Y Yang X Zhao L Wen S Liu Y. Association Between Polycystic Ovary Syndrome and Gut Microbiota. PloS One (2016) 11(4):e0153196. doi: 10.1371/journal.pone.0153196 27093642 PMC4836746

[36] Dou L Zheng Y Li L Gui X Chen Y Yu M. The Effect of Cinnamon on Polycystic Ovary Syndrome in a Mouse Model. Reprod Biol Endocrinol (2018) 16(1):99. doi: 10.1186/s12958-018-0418-y 30340496 PMC6194596

[37] Furat Rencber S Kurnaz Ozbek S Eraldemir C Sezer Z Kum T Ceylan S. Effect of Resveratrol and Metformin on Ovarian Reserve and Ultrastructure in PCOS: An Experimental Study. J Ovarian Res (2018) 11(1):55. doi: 10.1186/s13048-018-0427-7 29958542 PMC6025739

[38] Ryan GE Malik S Mellon PL. Antiandrogen Treatment Ameliorates Reproductive and Metabolic Phenotypes in the Letrozole-Induced Mouse Model of PCOS. Endocrinology (2018) 159(4):1734–47. doi: 10.1210/en.2017-03218 PMC6097580 29471436

[39] Boland NI Humpherson PG Leese HJ Gosden RG. Characterization of Follicular Energy Metabolism. Hum Reprod (1994) 9(4):604–9. doi: 10.1093/oxfordjournals.humrep.a138557 8046010

[40] Tu HY Lei XC Huo P Le JH Zhang S. Energy Demand and Its Regulatory Mechanism During Folliculogenesis. Zhongguo Yi Xue Ke Xue Yuan Xue Bao (2019) 41(3):408–14. doi: 10.1210/en.2016-1608 31282338

[41] Harlow CR Winston RM Margara RA Hillier SG. Gonadotrophic Control of Human Granulosa Cell Glycolysis. Hum Reprod (1987) 2(8):649–53. doi: 10.1093/oxfordjournals.humrep.a136609 3437044

[42] Zhang Y Liu L Yin TL Yang J Xiong CL. Follicular Metabolic Changes and Effects on Oocyte Quality in Polycystic Ovary Syndrome Patients. Oncotarget (2017) 8(46):80472–80. doi: 10.18632/oncotarget.19058 PMC5655213 29113318

[43] Zhang S Tu H Yao J Le J Jiang Z Tang Q. Combined Use of Diane-35 and Metformin Improves the Ovulation in the PCOS Rat Model Possibly *via* Regulating Glycolysis Pathway. Reprod Biol Endocrinol (2020) 18(1):58. doi: 10.1186/s12958-020-00613-z 32493421 PMC7268382

[44] Caldwell AS Eid S Kay CR Jimenez M McMahon AC Desai R. Haplosufficient Genomic Androgen Receptor Signaling is Adequate to Protect Female Mice From Induction of Polycystic Ovary Syndrome Features by Prenatal Hyperandrogenization. Endocrinology (2015) 156(4):1441–52. doi: 10.1210/en.2014-1887 25643156

[45] Caldwell ASL Edwards MC Desai R Jimenez M Gilchrist RB Handelsman DJ. Neuroendocrine Androgen Action is a Key Extraovarian Mediator in the Development of Polycystic Ovary Syndrome. Proc Natl Acad Sci U S A (2017) 114(16):E3334–E43. doi: 10.1073/pnas.1616467114 PMC5402450 28320971

[46] Ma Y Andrisse S Chen Y Childress S Xue P Wang Z. Androgen Receptor in the Ovary Theca Cells Plays a Critical Role in Androgen-Induced Reproductive Dysfunction. Endocrinology (2017) 158(1):98–108. doi: 10.1210/en.2016-1608 27841936 PMC5412974

[47] Wang D Weng Y Zhang Y Wang R Wang T Zhou J. Exposure to Hyperandrogen Drives Ovarian Dysfunction and Fibrosis by Activating the NLRP3 Inflammasome in Mice. Sci Total Environ (2020) 745:141049. doi: 10.1016/j.scitotenv.2020.141049 32758727

[48] Nejabati HR Samadi N Shahnazi V Mihanfar A Fattahi A Latifi Z. Nicotinamide and its Metabolite N1-Methylnicotinamide Alleviate Endocrine and Metabolic Abnormalities in Adipose and Ovarian Tissues in Rat Model of Polycystic Ovary Syndrome. Chem Biol Interact (2020) 324:109093. doi: 10.1016/j.cbi.2020.109093 32298659

[49] Song S Tan Y. Expression of FKBP52 in the Ovaries of PCOS Rats. Int J Mol Med (2019) 43(2):868–78. doi: 10.3892/ijmm.2018.3998 PMC6317667 30483787

[50] Yao K Bian C Zhao X. Association of Polycystic Ovary Syndrome With Metabolic Syndrome and Gestational Diabetes: Aggravated Complication of Pregnancy. Exp Ther Med (2017) 14(2):1271–76. doi: 10.3892/etm.2017.4642 PMC5526116 28810587

[51] Dunaif A. Insulin Resistance and the Polycystic Ovary Syndrome: Mechanism and Implications for Pathogenesis. Endocr Rev (1997) 18(6):774–800. doi: 10.1210/edrv.18.6.0318 9408743

[52] Macut D Bjekić-Macut J Rahelić D Doknić M. Insulin and the Polycystic Ovary Syndrome. Diabetes Res Clin Pract (2017) 130:163–70. doi: 10.1016/j.diabres.2017.06.011 28646699

[53] Zhang Y Zhou G Peng Y Wang M Li X. Anti-Hyperglycemic and Anti-Hyperlipidemic Effects of a Special Fraction of Luohanguo Extract on Obese T2DM Rats. J Ethnopharmacol (2020) 247:112273. doi: 10.1016/j.jep.2019.112273 31586692

