# Supplementary material for: Mogroside V Improves Follicular Development and Ovulation in Young-Adult PCOS Rats Induced by Letrozole and High-Fat Diet Through Promoting Glycolysis
Source: Front Endocrinol (Lausanne). 2022 Mar 28;13:838204. doi: 10.3389/fendo.2022.838204 (PMC8995474; doi:10.3389/fendo.2022.838204)
Supplement: Supplementary file 1 [file DataSheet_1.zip › Supplemental Table S1.DOCX]

**Supplemental Table S1. The antibodies of this study**

| Antibodies | Cat. no. | Company | Country | IHC | WB |
| --- | --- | --- | --- | --- | --- |
| HK2 | #2867 | Cell Signaling Technology, Inc. | Danvers, Massachusetts, USA | 1:400 | 1:1000 |
| PKM2 | #4053 | Cell Signaling Technology, Inc. | Danvers, Massachusetts, USA | 1:500 | 1:1000 |
| LDHA | #3558 | Cell Signaling Technology, Inc. | Danvers, Massachusetts, USA | 1:300 | 1:1000 |
| β-tubulin | No: 66240-1-Ig | Protein Tech Group Inc. | Chicago, USA |  | 1:3000 |
| horseradish peroxidase-conjugated goat anti-rabbit IgG | SA00001-2 | Protein Tech Group Inc. | Chicago, USA | 1:200 | 1:5000 |
| biotin-conjugated affinipure goat anti-rabbit IgG | SA00004-2 | Protein Tech Group Inc. | Chicago, USA | 1:200 |  |
