# Supplementary material for: Mogroside V Improves Follicular Development and Ovulation in Young-Adult PCOS Rats Induced by Letrozole and High-Fat Diet Through Promoting Glycolysis
Source: Front Endocrinol (Lausanne). 2022 Mar 28;13:838204. doi: 10.3389/fendo.2022.838204 (PMC8995474; doi:10.3389/fendo.2022.838204)
Supplement: Supplementary file 1 [file DataSheet_1.zip › Supplemental Table S2.DOCX]

**Supplemental Table S2. The key reagents of this study**

| Reagents | Cat. No. | Company | Country |
| --- | --- | --- | --- |
| Letrozole | 190605KG | Heng Rui Pharmaceutical Company | Lianyungang, Jiangsu, China |
| Mogroside V | MOV04-18022504 | Guilin Layn Natural Ingredients Corp. | Guilin, Guangxi, China |
| TransScript® One-Step gDNA Removal and cDNA Synthesis SuperMix Kit | AT311-02 | TransGen Biotech | Beijing, China |
| 2 × Universal SYBR Green Fast qPCR Mix kit | RK21203 | ABclonal Technology Co., Ltd. | Wuhan, China |
| Wright’s–Giemsa Stain solution | G1020 | Beijing Solarbio Science & Technology Co.,Ltd | Beijing, China |
| 20 × Metal Enhanced DAB Substrate Kit | DA1015 | Beijing Solarbio Science & Technology Co.,Ltd | Beijing, China |
| BCA Protein Assay Kit | CW0014S | Beijing ComWin Biotech Co., Ltd. | Beijing, China |
| eECL Western Blot Kit | CW0049M | Beijing ComWin Biotech Co., Ltd. | Beijing, China |
| SDS-PAGE Gel Kit | CW0022S | Beijing ComWin Biotech Co., Ltd. | Beijing, China |
| Trizol reagent | 15596026 and 15596018 | Thermo Fisher Scientific | Waltham, USA |
